# Fusion dual-tracer SPECT-based hepatic dosimetry predicts outcome after radioembolization for a wide range of tumour cell types

**DOI:** 10.1007/s00259-015-3048-z

**Published:** 2015-04-28

**Authors:** Marnix G. E. H. Lam, Arjun Banerjee, Michael L. Goris, Andrei H. Iagaru, Erik S. Mittra, John D. Louie, Daniel Y. Sze

**Affiliations:** Division of Interventional Radiology, Stanford University, Stanford, CA USA; Department of Radiology and Nuclear Medicine, University Medical Center Utrecht, Heidelberglaan 100, 3584 CX Utrecht, The Netherlands; Division of Nuclear Medicine and Molecular Imaging, Stanford University, Stanford, CA USA

**Keywords:** Radioembolization, Treatment planning, Functional imaging, Partition model, SPECT

## Abstract

**Purpose:**

Fusion dual-tracer SPECT imaging enables physiological rather than morphological voxel-based partitioning and dosimetry for ^90^Y hepatic radioembolization (RE). We evaluated its prognostic value in a large heterogeneous cohort of patients with extensive hepatic malignancy.

**Methods:**

A total of 122 patients with primary or secondary liver malignancy (18 different cell types) underwent SPECT imaging after intraarterial injection of ^99m^Tc macroaggregated albumin (TcMAA) as a simulation of subsequent ^90^Y microsphere distribution, followed by administration of an excess of intravenous ^99m^Tc-labelled sulphur colloid (TcSC) as a biomarker for functional liver, and a second SPECT scan. TcMAA distribution was used to estimate ^90^Y radiation absorbed dose in tumour (*D*_T_) and in functional liver. Laboratory and clinical follow-up were recorded for 12 weeks after RE, and radiographic responses according to (m)RECIST were evaluated at 3 and 6 months. Dose–response relationships were determined for efficacy and toxicity.

**Results:**

Patients were treated with a median of 1.73 GBq activity of resin microspheres (98 patients) or glass microspheres (24 patients), in a whole-liver approach (97 patients) or a lobar approach (25 patients). The objective response rate was 41 % at 3 months and 48 % at 6 months. Response was correlated with *D*_T_ (*P* < 0.01). Median overall survival was 10.1 months (95 % confidence interval 7.4 – 12.8 months). Responders lived for 36.0 months compared to 8.7 months for nonresponders (*P* < 0.01). Stratified for tumour cell type, *D*_T_ was independently associated with survival (*P* < 0.01). Absorbed dose in functional liver was correlated with toxicity grade change (*P* < 0.05) and RE-induced liver disease (*P* < 0.05).

**Conclusion:**

Fusion dual-tracer SPECT imaging offers a physiology-based functional imaging tool to predict efficacy and toxicity of RE. This technique can be refined to define dosing thresholds for specific tumour types and treatments, but appears generally predictive even in a heterogeneous cohort.

**Electronic supplementary material:**

The online version of this article (doi:10.1007/s00259-015-3048-z) contains supplementary material, which is available to authorized users.

## Introduction

Treatment planning is critical to the success of ^90^Y hepatic radioembolization (RE) [1]. Safety and efficacy are contingent upon inhomogeneous intrahepatic distribution of radioactive microspheres to achieve tumoricidal doses without serious hepatic injury. Current standard methods for activity calculation for both resin and glass microspheres were largely validated on empirical grounds [2–8]. Although these early dose ranging studies were sufficient to show promising results and an acceptable toxicity profile, imperfect response rates and occasional severe toxicities continue to drive investigators towards optimization of RE treatment planning [9].

Recent proposals to improve treatment planning are based on morphological partition modelling using CT or MRI to delineate the anatomical borders of tumours within the liver volume, combined with SPECT imaging to calculate the activity distribution within these target volumes [10, 11]. These methods incorporate dosimetric parameters such as tumour and normal liver absorbed doses in the activity calculations, and seem to be feasible and accurate in patients with a limited number of clearly demarcated tumours, mostly hepatocellular carcinomas (HCC) [12]. However, in the presence of diffuse, infiltrative HCC or myriad heterogeneous metastases, anatomical partition modelling is prone to significant error [1]. These partition methods are therefore difficult to standardize in clinical practice.

We proposed a new partition method using physiological parameters only: a segmentation tool based on a dual-tracer SPECT technique, combining ^99m^Tc macroaggregated albumin (TcMAA) SPECT for simulation of ^90^Y activity distribution, and ^99m^Tc-sulphur colloid (TcSC) SPECT for identifying functional liver parenchyma [13]. This method obviates the need to delineate the different compartments by anatomical imaging and is automated, fast, and objective. In a homogeneous cohort of 25 patients with metastatic colorectal carcinoma (mCRC), we found that the calculated absorbed doses to the tumour compartment (*D*_T_) and to the functional liver compartment (*D*_FL-TOT_) are significantly correlated with efficacy and toxicity. In the present study, we broadened validation to a large heterogeneous cohort of patients with many different tumour cell types of different anatomical and vascular characteristics.

## Materials and methods

### Patient selection

Between June 2004 and September 2011, 247 consecutive patients underwent intraarterial TcMAA imaging as part of their RE preparatory work-up. Of these, 184 underwent intravenous TcSC imaging. In 38 patients, mismatch in injection positions of TcMAA and ^90^Y jeopardized the accuracy of distribution simulation and these patients were excluded. In addition, 24 patients who received treatment in two staged sessions were excluded because pretreatment TcMAA was injected nonstaged and nonselectively. A total of 122 patients (68 men, 54 women; median age 62 years, range 25 – 92 years) were included in the analysis, including 25 previously studied patients [13]. Baseline, procedural, and follow-up data were collected prospectively as standard of care, and retrospectively analysed for this study. Data were handled in accordance with the Health Insurance Portability and Accountability Act. The institutional review board approved the study. Table 1 summarizes the patient characteristics.

**Table 1:** Demographics, baseline characteristics and oncological histories of the cohort

Characteristic	Value
Sex (male/female), *n*	68/54
Age (years), median (range)	62 (25–92)
Tumour cell type, *n* (%)	
Hepatocellular carcinoma	26 (21.3)
Cholangiocarcinoma	18 (14.7)
Metastatic neuroendocrine carcinoma	20 (16.4)
Metastatic colorectal carcinoma	29 (23.8)
Other^a^	29 (23.8)
Previous systemic treatment, *n* (%)	
Cytotoxic chemotherapy	74 (60.7)
Bevacizumab	36 (29.5)
Sorafenib	12 (9.8)
Anti-EGFR agents	18 (14.8)
Previous liver-directed treatment, *n* (%)	
Transarterial (chemo)embolization	27 (22.1)
Partial liver resection	26 (21.3)
Radiofrequency ablation	18 (14.8)
External beam radiation therapy^b^	8 (6.6)
Hepatic radioembolization	7 (5.7)
ECOG performance status, *n* (%)	
0	54 (44.3)
1	58 (47.5)
2	10 (8.2)
Extrahepatic disease, *n* (%)	61 (50)
Lung	37 (30.3)
Lymph nodes	16 (13.1)
Bone	10 (8.2)
Estimated liver tumour involvement (%), median (range)	25 (5–70)
Maximum tumour diameter (mm), median (range)	52 (15–133)
Liver cirrhosis, *n* (%)	22 (18)
Microspheres, *n* (%)	
Resin	98 (80.3)
Glass	24 (19.7)
Treatment, *n* (%)	
Whole liver	97 (79.5)
Lobar	25 (20.5)
Administered activity (GBq), median (range)	1.73 (0.43–6.21)

*EGFR* epidermal growth factor receptor

^a^Includes sarcoma (five patients), melanoma (four), renal cell carcinoma (three), pancreatic adenocarcinoma (three), oesophageal carcinoma (three), ovarian carcinoma (one), urothelial carcinoma (one), small-cell lung carcinoma (one), lymphoma (one), cervical carcinoma (one), thymic carcinoma (two), breast carcinoma (two), ampullary carcinoma (one), and gastronintestinal junction carcinoma (one)

^b^The mean radiation absorbed dose to the liver was determined by dose–volume histogram analysis. All mean liver doses >1 Gy were included

### Radioembolization

Activity calculations and treatments were performed according to international consensus guidelines [14–16]. Resin microspheres (SIR-Spheres; SIRTex Medical Ltd., North Sydney, Australia) were used to treat 98 patients (80.3 %). The prescribed activity was calculated based on body surface area (BSA) and estimated tumour liver involvement (LI; median 25 %, range 5–70 %), where prescribed activity (GBq) = BSA (m^2^) − 0.2 + LI [12]. Glass microspheres (TheraSphere; BTG, Inc., Farnham, UK) were used to treat 24 patients, applying a medical internal radiation dosimetry (MIRD) method to prescribe a desired target territory absorbed dose of 90 – 120 Gy [17]. Hepatopulmonary shunting was compensated for by the recommended activity adjustments [17, 12].

Clinical and laboratory follow-up were performed 2, 4, 8 and 12 weeks after treatment, and at intervals prescribed by the medical oncologist thereafter. Toxicity was graded according to National Cancer Institute common toxicity criteria for adverse events (NCI-CTCAE v4.03). Follow-up imaging replicating the pretreatment modality was used for response analysis according to modified Response Evaluation Criteria in Solid Tumors (mRECIST) [18] for HCC and RECIST 1.1 for other tumour types, with a focus on liver response only [19]. One blinded reviewer performed over-reads of all clinical interpretations.

### Imaging procedures

As described previously [13], routine SPECT was performed after intraarterial administration of a small TcMAA dose (37 MBq). Without moving the patient, an excess of TcSC (185 MBq) was administered intravenously, and SPECT was repeated after a 5-min delay. SPECT data were acquired on a dual-head Infinia Hawkeye 4 gamma camera (GE Healthcare, Waukesha, WI), with a 64 × 64 matrix (voxel size 0.884 mm^3^), 130–150 keV energy window, low-energy high-resolution collimator and 120 projections (15 s per projection) over a 360° full circular orbit.

### Image processing and analysis

**Fig. 1:**
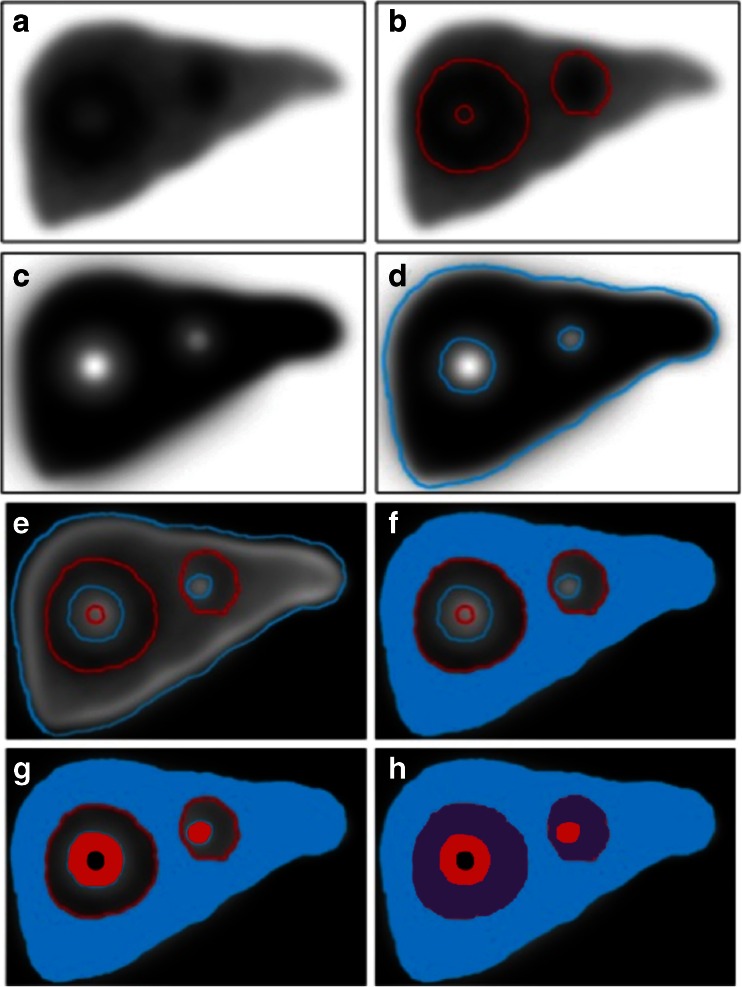
A threshold applied to the TcMAA SPECT image (**a**) defines the MAA-positive volume (**b**), and to the ^99m^Tc-sulphur colloid (SC) SPECT (**c**) defines the SC-positive volume (**d**). Coregistration of the two SPECT scans results in four compartments: **f** unirradiated functional liver (*V*_FL-UN_), MAA-negative, SC-positive (*blue*), **g** tumour (*V*_T_), MAA-positive SC-negative (*red*), **h** irradiated functional liver (*V*_FL-IR_), MAA-positive SC-positive (*purple*), and tumour necrosis (*V*_NULL_), MAA-negative SC-negative (*black*)

**Fig. 2:**
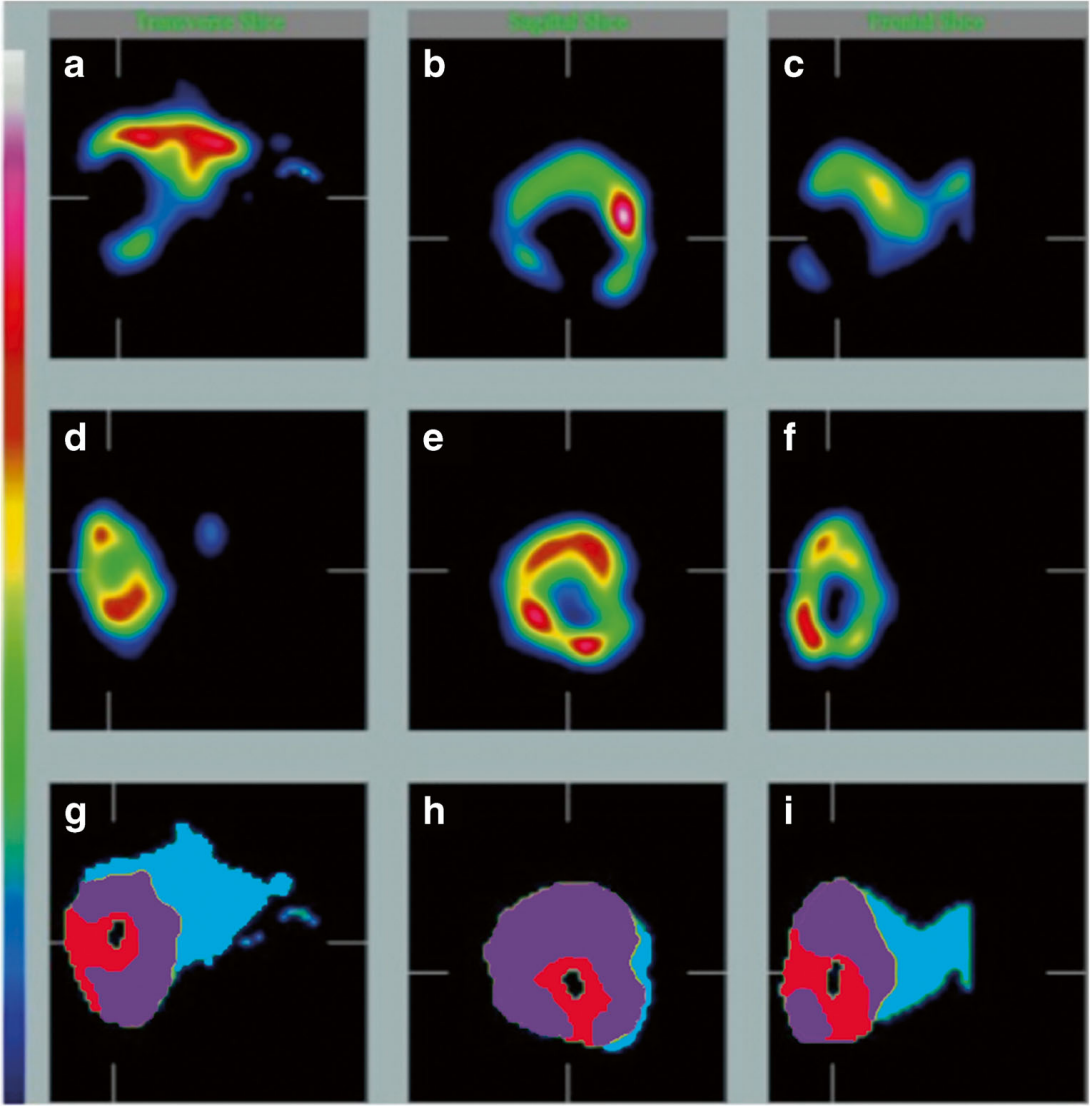
Functional liver tissue defined by TcSC SPECT images in three orientations (axial, sagittal, coronal, **a**–**c**), simulated ^90^Y distribution defined by TcMAA SPECT images in three orientations (**d**–**f**), and the coregistered fused SPECT images in three orientations (**g**–**i**), resulting in the definition of four different compartments: tumour (*red*), tumour necrosis (*black*), irradiated functional liver (*purple*), and unirradiated functional liver (*blue*)

**Fig. 3:**
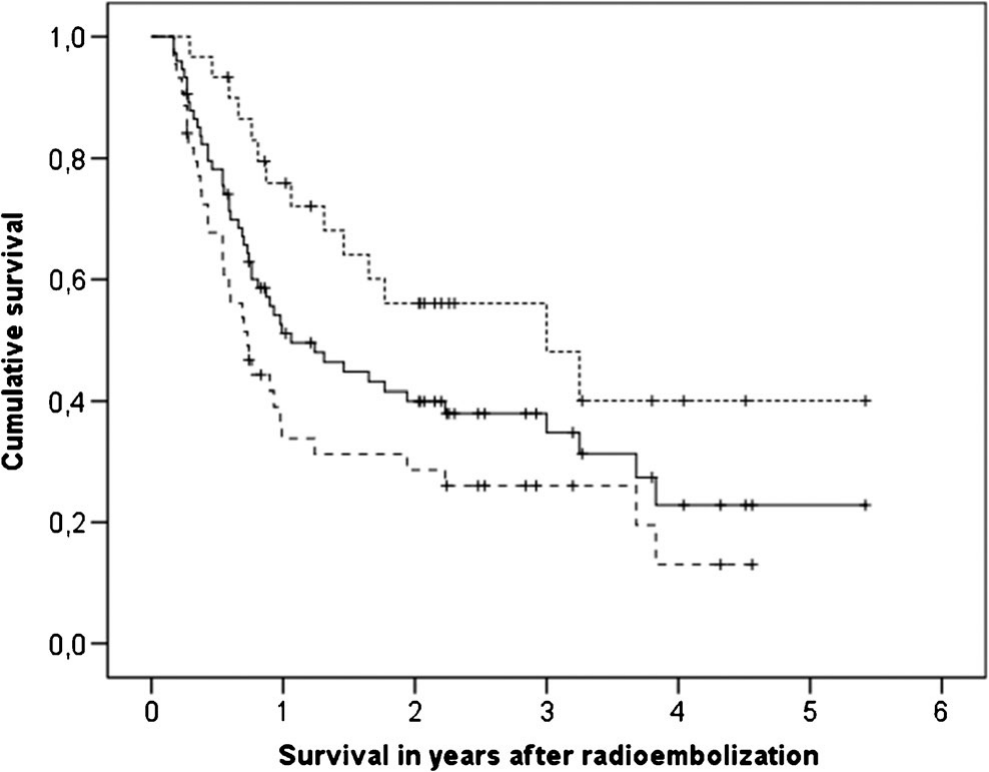
Kaplan-Meier survival curves for patients with an objective response at 3 months (*upper dotted line*), patients without response (*lower dashed line*), and all patients (*middle solid line*). Median survival in responders was 36 months versus only 8.7 months in nonresponders (*P* = 0.003); stratified according to tumour cell type, this was still significant (*P* = 0.011)

Data were reconstructed using filtered back projection and a Butterworth postreconstruction filter (Fc 0.23; order 6), using Segami software (Segami, Columbia, MD). From the TcMAA SPECT images (Fig. 1a), a three-dimensional tumour map (Fig. 1b) was generated by applying a threshold including all voxels with 10 % or more of the maximum TcMAA per voxel, using software programmed in IDL 6.1 (Exelis, Inc., McLean, VA). This threshold was chosen after comparison between 5–30 %. Corrected TcSC images (Fig. 1c) were then calculated by subtracting TcMAA images (Fig. 1a) from the TcSC images. A map of functional liver (Fig. 1d) was produced by applying a 10 % threshold to the corrected TcSC images. Fusing the TcMAA and TcSC maps (Fig. 1e) resulted in hepatic segmentation into four compartments: (1) the unirradiated functional liver compartment (*V*_FL-UN_, TcSC-positive only; Fig. 1f); (2) the tumour compartment (*V*_T_, TcMAA-positive only; Fig. 1g); (3) the overlap area, the irradiated functional liver compartment (*V*_FL-IR_, both TcSC- and TcMAA-positive; Fig. 1h) which typically represented the marginal zone that included hypervascular rims; and (4) the null compartment (*V*_NULL_, both TcSC- and TcMAA-negative) which included central necrosis, major vascular structures, cysts, etc. The total functional liver compartment or *V*_FL-TOT_ was defined as *V*_FL-IR_ plus *V*_FL-UN_, and the total liver volume *V*_TOTAL LIVER_ was calculated as *V*_T_ plus *V*_FL-TOT_. The process of fusion of the TcSC and TcMAA images to define the four liver compartments is shown in Fig. 2.

Voxel counts were converted into volumes, and deposited TcMAA activities were converted into compartment absorbed doses using the MIRD formula [20]:

Compartment absorbed dose = (compartment TcMAA activity/total TcMAA activity) × administered activity (GBq) × 1.029^−1^ (kg/L) × volume^−1^ (L) × 50

where ‘compartment TcMAA activity’ is the measured TcMAA activity in a compartment, ‘total TcMAA activity’ is the total TcMAA activity in the liver, 1.029 kg/L is the estimated specific density of hepatic tissue, ‘volume’ is the calculated volume of the compartment (using a 10 % threshold as described above), and ‘50’ is the conversion factor for ^90^Y from GBq/kg to Gy. Below the 10 % threshold, calculated *D*_FL-UN_ to the unirradiated functional liver was simplified as zero. Calculated absorbed dose in the other three compartments (*D*_T_, *D*_FL-TOT_, *D*_FL-IR_), volumes of each compartment (*V*_T_, *V*_FL-TOT_, *V*_FL-UN_ and *V*_FL-IR_), ratios between parameters, and fractional volumes were evaluated for dose–response relationships with regard to efficacy and toxicity (Supplementary Tables 1 and 2).

### Statistical analysis

A commercial software package was used for statistical analysis (SPSS for Windows, version 19.0; SPSS Inc, Chicago, IL). The normality of the distributions of continuous variables was tested using the Kolmogorov-Smirnov test. Nonparametric tests were used to compare groups (chi-squared test for categorical variables, Mann-Whitney or Kruskal-Wallis one-way ANOVA for continuous variables). Survival was evaluated using Kaplan-Meier curves. Stratification was performed for tumour cell type with the log rank test for comparison pooled over the strata. Multivariate survival analysis was performed with a Cox proportional hazards model using the conditional step forward method (stepwise probability: entry 0.05, removal 0.10). A *P* value <0.05 was considered statistically significant.

## Results

The 122 patients were treated with a median of 1.73 GBq resin microspheres (98 patients) or glass microspheres (24 patients), in a whole-liver distribution (97 microspheres) or lobar distribution (25 microspheres; Table 1). The majority of patients treated with glass microspheres had HCC (22 of 24 patients). The median administered activity of glass microspheres was 3.37 GBq compared to 1.64 GBq for resin microspheres (*P* < 0.001). The median tumour absorbed dose (*D*_T_) in the total cohort was 36.3 Gy, and the median functional liver absorbed dose (*D*_FL-TOT_) was 29.7 Gy (Table 2). This was correlated significantly with tumour cell type (*P* < 0.001). Patients with HCC had a much higher median *D*_T_ (109.7 Gy) and *D*_FL-TOT_ (55.1 Gy) than those with other cell types, mostly because treatment with higher activity of glass microspheres resulted in higher *D*_T_ (116 Gy versus 32.7 Gy; *P* < 0.001) and *D*_FL-TOT_ (57.1 Gy versus 27.3 Gy; *P* < 0.001). However, independent of the type of microspheres used, HCC also had a higher *D*_T_/*D*_FL-TOT_ ratio (median 1.8; *P* = 0.02; Table 2), probably reflecting hypervascularity and focal tumours.

**Table 2:** Treatment parameters according to tumour cell type

Tumour cell type	No. of patients	Maximum tumour diameter (mm)	Calculated absorbed doses (Gy)	
			Tumour, *D*_T_	Functional liver, *D*_FL-TOT_
Hepatocellular carcinoma	26	56	109.7	55.1
Cholangiocarcinoma	18	76	35	24.9
Neuroendocrine carcinoma	20	56	24.2	23.7
Colorectal carcinoma	29	46	33.3	27.8
Other^a^	29	44	33.6	29.8
All tumour types	122	52	36.3	29.7
*P* values (between groups)^b^	NA	0.471	<0.001	<0.001

Variables are reported as medians

^a^Tumour cell types in this group are listed in Table 1 footnote a

^b^Nonparametric Kruskal-Wallis one-way ANOVA for multiple pairwise comparison

Of the 122 patients, 74 were evaluable for response at 3 months (18 died before follow-up imaging, 5 had inadequate baseline imaging, and 25 did not have adequate follow-up imaging), and 44 were evaluable at 6 months (48 died, 2 inadequate baseline imaging, 28 inadequate follow-up imaging). The objective response rates (complete plus partial responses) were 41 % at 3 months and 48 % at 6 months (Table 3). Response at 3 months was correlated only with *D*_T_ in the univariate analysis (*P* = 0.026) and the multivariate analysis (*P* = 0.004). Other significant dosimetry parameters (Supplementary Table 1) were strongly interrelated and were thus excluded. At 6 months, only a trend was found for *D*_T_ in the univariate analysis (*P* = 0.069). At 3 months, responders had a median *D*_T_ of 60.1 Gy versus 32.7 Gy for nonresponders (*P* = 0.026), and at 6 months 60.5 Gy versus 29.3 Gy (*P* = 0.069). The median survival (from treatment) in responders at 3 months was 36.0 months, and in nonresponders was 8.7 months (*P* = 0.003; stratified according to tumour cell type: *P* = 0.011; Fig. 3).

**Table 3:** Treatment outcome according to tumour cell type

Tumour cell type	Survival (months), median (95 % confidence interval)		Response, *n* (%)^a^		Grade 3 or 4 toxicity, *n* (%)	REILD
	From treatment	From diagnosis	At 3 months	At 6 months		
Hepatocellular carcinoma	8 (2.4–13.6)	32.4 (24.9–44)	9/15 (60)	7/10 (70)	10/22 (46)	2
Cholangiocarcinoma	5.7 (2.0–9.4)	21 (12.6–29.4)	2/11 (18)	2/5 (40)	2/16 (13)	1
Neuroendocrine carcinoma	Not reached	Not reached	8/14 (57)	6/13 (46)	1/19 (5)	0
Colorectal carcinoma	10.8 (6.1–15.5)	37.9 (30–45.8)	5/19 (26)	3/10 (30)	5/28 (18)	1
Other^b^	8.3 (6–10.6)	32.8 (18.7–46.9)	6/15 (40)	3/6 (50)	6/26 (23)	1
All tumour types	10.1 (7.4–12.8)	37.7 (31.1–44.3)	30/74 (41)	21/44 (48)	24/111 (22)	5
Evaluable patients	122	122	74	44	111	111
*P* values (between groups)^c^	<0.001	<0.001	0.098	0.494	0.022	NA

*REILD* radioembolization-induced liver disease

^a^Response includes complete response and partial response by RECIST or mRECIST, as described in the text

^b^Tumour cell types in this group are listed in Table 1 footnote a

^c^Log-rank test for survival comparison between groups; nonparametric chi-squared test for response and toxicity comparison between groups

**Fig. 4:**
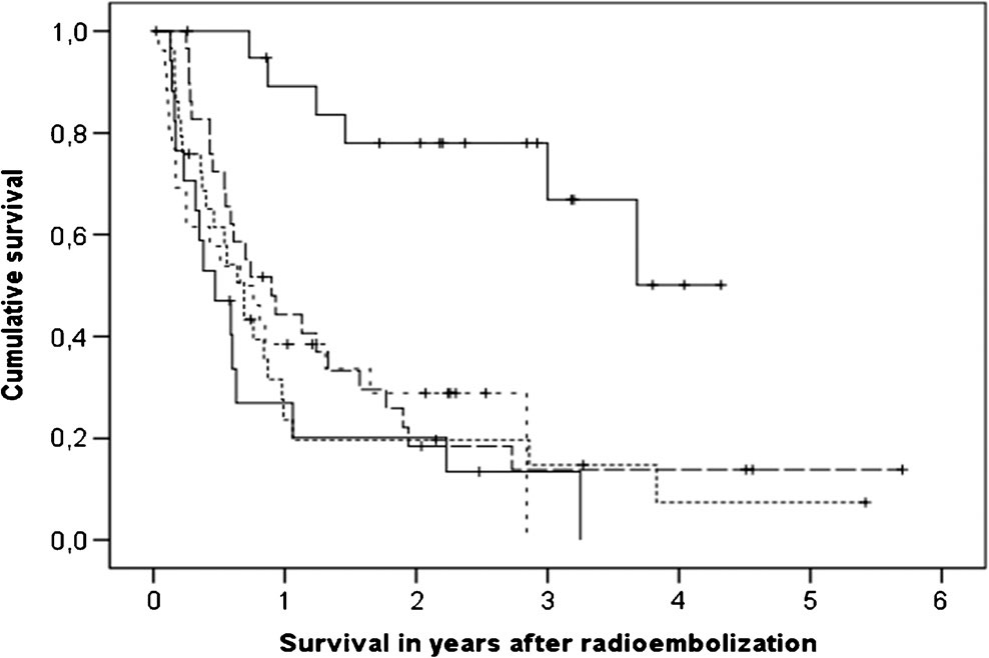
Kaplan-Meier survival curves for patients with hepatocellular carcinoma (*short dashes*), cholangiocarcinoma (*lower solid line*), metastatic neuroendocrine carcinoma (*upper solid line*), metastatic colorectal carcinoma (*long dashes*), and miscellaneous metastatic malignancies (*dotted line*)

The overall median survival from treatment was 10.1 months (95 % confidence interval 7.4 – 12.8 months), and from diagnosis 37.7 months (95 % confidence interval 31.1 – 44.3 months; Table 3). At the time of writing 34 patients were still alive with a median follow-up of 27.1 months. Survival from treatment (*P* < 0.001) and survival from diagnosis (*P* < 0.001) were dependent on tumour cell type (Fig. 4). Patients with HCC were selected for treatment with RE rather than chemoembolization if they had very large tumours (>8 cm), infiltrative disease, macrovascular invasion, and/or had failed prior chemoembolization (Table 2), resulting in expected poor overall survival after treatment (Table 3). Of 26 HCC patients for example, 22 had underlying liver cirrhosis with significantly worse liver function, and 5 had main portal vein thrombosis. Stratified according to tumour cell type, *D*_T_ was correlated with survival after treatment in the univariate analysis (*P* = 0.004) and multivariate analysis (*P* = 0.004). Only response, tumour cell type, and *D*_T_ were correlated with survival.

**Fig. 5:**
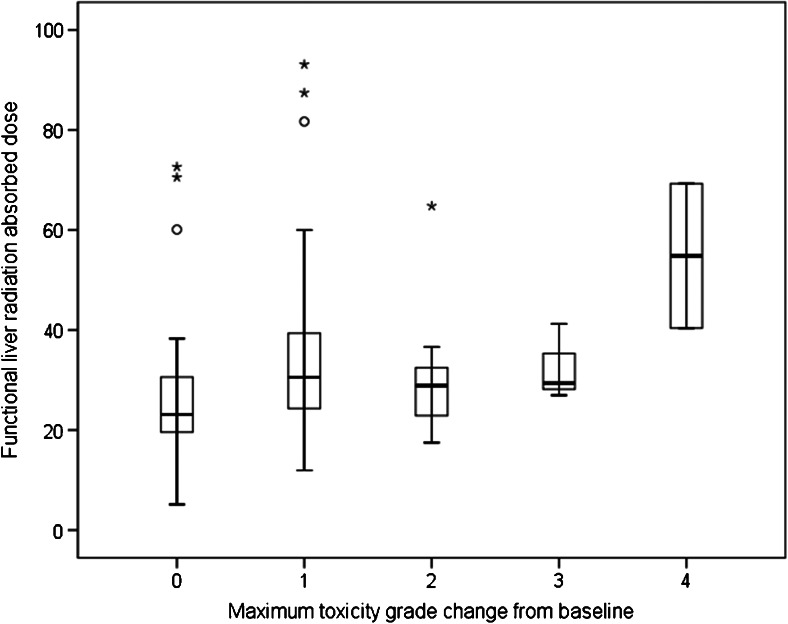
Maximum change in toxicity grade from baseline during follow-up was significantly associated with the radiation absorbed dose to functional liver tissue (*D*_FL-TOT_). Medians, and first and third quartiles are indicated by the boxes (central line, and lower and upper border, respectively), and minimum and maximum by the T-bars (*circles* outliers, >1.5 times interquartile range; *asterisks* extreme outliers, >3 times interquartile range)

Treatment-related adverse events were usually mild, expected, and limited to grade 1/2 toxicity such as nausea, abdominal discomfort and fatigue. Laboratory values changed as expected (Supplementary Fig. 1). Grade 3/4 toxicity occurred in 24 patients, most of whom had preexisting grade 1/2 toxicity. Grade 4 toxicity included increases in bilirubin (two patients) and aspartate aminotransferase (AST, one patient). Absolute grade 3/4 toxicity was related to tumour cell type (with a 46 % incidence among those with HCC; *P* = 0.022), liver cirrhosis (*P* = 0.002), baseline AST (*P* = 0.002), and alanine aminotransferase (ALT, *P* = 0.004). However, *D*_FL-TOT_ was the strongest (*P* = 0.010) and most comprehensive dosimetry parameter associated with an increase in toxicity grade (Supplementary Table 2 and Fig. 5). Other parameters associated with an increase in toxicity grade included percent liver involvement (*P* = 0.038), baseline performance status (*P* = 0.017), and prior liver resection (*P* = 0.020).

RE-induced liver disease (REILD), defined as liver failure, hyperbilirubinaemia and ascites in the absence of tumour progression, occurred in five patients (4.5 %). *D*_FL-TOT_ was associated with REILD (*P* = 0.011). In addition, pretreatment AST (*P* = 0.006) and ALT (*P* = 0.035), performance status (*P* = 0.038), and previous RE (*P* = 0.031) were also associated with REILD. Interestingly, three of the five patients had received previous radiation therapy to the liver, either RE or external beam radiation therapy (EBRT). The median *D*_FL-TOT_ in patients with REILD was 41.2 Gy, compared to 27.9 Gy in the other patients (*P* = 0.011). However, corrected for previous radiation exposure calculated by dose–volume histogram (DVH) analysis [21], the cumulative *D*_FL-TOT_ was 64.8 Gy versus 27.9 Gy (*P* = 0.009). Five of 13 patients (38.5 %) with cumulative *D*_FL-TOT_ >60 Gy had REILD (*P* < 0.001). None had progressive disease during follow-up (partial response in three patients, stable disease in one patient, no imaging in one patient with biopsy-proven venoocclusive disease), but median survival in these five patients was only 84 days.

The calculated *D*_T_/*D*_FL-TOT_ ratio, analogous to a physiological tumour to normal (T/N) ratio, was <1 in 41 of the 122 patients (33.6 %). This was encountered significantly more frequent in patients with secondary malignancy (33 of the 41 patients; *P* = 0.009), often in patients with large overlap areas that were positive for both TcMAA and TcSC. In contrast to anatomical segmentation methods these overlapping areas were included in the normal liver segment, which leads to generally lower T/N ratios. Efficacy and toxicity parameters were not found to be associated with *D*_T_/*D*_FL-TOT_ <1. *D*_T_/*D*_FL-TOT_ depended on tumour cell type (Table 2), but was not correlated with response at 3 and 6 months, toxicity, toxicity grade increase, or REILD.

Of the 122 patients, 24 were treated with glass microspheres (22 with HCC). Compared to patients treated with resin microspheres (4 of 98 with HCC), patients treated with glass microspheres had a statistically significant worse baseline ECOG performance status and liver function with 75 % known to have liver cirrhosis versus 4 % (*P* < 0.001). They were treated with higher activities, which resulted in higher *D*_T_ and *D*_FL-TOT_. Response rates at 3 months (64 % versus 35 %; *P* = 0.069) and 6 months (78 % versus 40 %; *P* = 0.064) showed a trend in favour of glass microspheres, but survival showed no difference (heavily biased by HCC). Response at 3 months was associated with prolonged survival in both groups (median 8.8 versus 39.1 months for resin, and 6.5 versus 19.8 months for glass; *P* = 0.005). With regard to toxicity, worse baseline characteristics and higher *D*_FL-TOT_ after treatment with glass microspheres did not result in any association between glass microspheres and increased toxicity, perhaps also because fewer patients received whole-liver treatment compared to those treated with resin microspheres (42 % versus 89 %; *P* < 0.001). Microsphere type (i.e. resin or glass) was included as a parameter in the multivariate analysis, but was not independently associated with efficacy or toxicity (see above). Liver cirrhosis was associated with worse baseline laboratory values, but did not result in increased toxicity per se (being associated with *absolute* toxicity grade, but not with *changes* in toxicity grade). As expected, patients with cirrhosis did have a worse survival than noncirrhotic patients (median 5.2 versus 10.8 months; *P* = 0.033).

## Discussion

Fusion TcMAA/TcSC SPECT imaging enables physiological partitioning of the liver for intrahepatic RE dosimetry. It allows calculation of the absorbed dose to the tumour and to the functional liver parenchyma, even in patients with extensive infiltrative and/or multifocal disease in whom anatomical imaging-based partitioning is not feasible [1]. Across a large variety of primary and metastatic liver tumours, we found that tumour dose *D*_T_ was correlated with objective response and overall survival, and functional liver dose *D*_FL-TOT_ was correlated with toxicity. This study clearly confirmed dose–response relationships in a large heterogeneous cohort of patients typical of the population treated by RE.

Previous partitioning methods based on morphological anatomical imaging have been applied on patients with limited disease [22, 23]. Garin et al. segmented the liver on TcMAA SPECT/CT images by semiautomatic generation of a volume of interest over the tumour on the SPECT images using an isocontour method to match the tumour on the CT images. In a preliminary report in 36 HCC patients they showed that a threshold *D*_T_ value of 205 Gy was predictive of response [10]. These findings were confirmed in the extended cohort of 71 patients. Dosimetry enabled treatment intensification with favourable clinical outcome in selected patients, especially in patients with large tumours and portal vein thrombosis [24]. Mazzaferro et al. also found a correlation between *D*_T_ and response in 52 HCC patients, albeit at a higher threshold value of 500 Gy using manual delineation of the tumour [25]. A maximum safety threshold for normal liver parenchyma of 70 Gy was advocated based on these data [26]. Although promising, anatomical partitioning is limited in clinical practice, mainly because the presence of extensive or diffuse malignancy is associated with substantial error. Also, these methods do not adequately account for the compartment that contains both tumour and functional liver directly surrounding the tumour.

In our study, *D*_T_ was also correlated with response and with survival. In addition, toxicity was associated with the functional liver dose *D*_FL-TOT_, a finding consistent with existing understanding of radiation hepatotoxicity [27]. Patients with REILD had a significantly higher *D*_FL-TOT_, often due to previous hepatic radiation exposure. Prior EBRT, as well as prior RE treatment of the same target volume, is known to increase RE toxicity [21, 28]. When DVH analysis and voxel-based fusion SPECT dosimetry were applied to our cohort, we found that cumulative *D*_FL-TOT_ was above 60 Gy in all patients with REILD. The probability of REILD was further increased by poor performance status and poor liver function at baseline.

The volume of the unirradiated part of the functional liver (*V*_FL-UN_) proved to be associated with toxicity grade change and REILD, and with survival. This mirrors the surgical tenet that sufficient functional liver must be preserved for a better outcome after resection. A future liver remnant (FLR) fraction larger than 20 – 30 % is recommended in patients with a normal liver, whereas a remnant of >40 % is recommended in patients with cirrhosis [29]. The surgical concept of FLR could be adapted for application to RE, with thresholds to be defined for both the minimum volume of and the maximum dose to ‘unirradiated functional liver’.

Our ratio *D*_T_/*D*_FL-TOT_ is distinctly different from the commonly cited T/N ratio. Our physiological definition of functional liver assigns marginal tissue to the ’normal’ compartment, which probably includes hypervascular rims and ill-defined tumours interspersed with functional parenchyma. In contrast, the more commonly used T/N ratio is based on anatomical segmentation, where ‘T’ probably includes some functional liver surrounding the tumour. Assigning these irradiated areas to ‘T’ will increase the T/N ratio in comparison to our physiological *D*_T_/*D*_FL-TOT_. Including the overlap area in *D*_FL-TOT_ resulted in more accurate prediction of toxicity as would be expected from the scintigraphic characteristics. Some investigators have advocated using the ratio to guide patient selection [30, 31], finding that the FDG PET response in mCRC lesions can be predicted using a cut-off T/N ratio of 1 [30]. However, this remains controversial, since another study in 58 mCRC patients found no correlation with response [32]. ‘T’ and ‘N’ compartments were defined by morphological imaging only, which may have contributed to the lack of correlation. Our method using physiological characterization showed that the ratio *D*_T_/*D*_FL-TOT_ was not correlated with efficacy or toxicity; rather, the actual values of *D*_T_ and *D*_FL-TOT_ were more predictive.

Based on the results of this study, some preliminary suggestions may be provided for prospective use of dual-tracer SPECT dosimetry. None of the patients with REILD had a *D*_FL-TOT_ <30 Gy from RE or a cumulative *D*_FL-TOT_ <60 Gy. This suggests an alternative strategy for dose prescription: to adjust the administered activity to keep *D*_FL-TOT_ below a risk threshold. Surprisingly, a cumulative *D*_FL-TOT_ of 30 – 60 Gy appeared to be well tolerated, but the risk of REILD if *D*_FL-TOT_ exceeded 60 Gy was 38 %. A *D*_T_ >32.7 Gy led to a 50 % objective response rate, irrespective of tumour cell type. It seems reasonable to aim for *D*_T_ >32.7 Gy if cumulative *D*_FL-TOT_ can be kept below 30 Gy. Under this proposal, of the 122 patients in our cohort, administered activity could potentially have been increased in 65 patients (53 %). Thirteen patients (11 %), including the five with REILD, would have required activity reduction to keep *D*_FL-TOT_ below 30 Gy. This strategy will need to be validated using a prospective protocol.

Threshold *D*_T_ and *D*_FL-TOT_ may prove to be different between glass and resin microspheres. These products differ in activities prescribed, specific activity, embolization effect, and often proportion of the liver treated. Dose–effect relationships for both efficacy and toxicity are influenced by these differences. Dose–effect relationships probably also differ for each tumour cell type and for different baseline liver function statuses. With additional experience, thresholds should be defined tailored to different tumour cell types and product used. However, even with our amalgamated heterogeneous cohort, significant dose–response relationships were identified.

The limitations of this study include its retrospective design and imperfect toxicity and efficacy analysis. Strong endpoints, such as REILD, had a low incidence, while weaker endpoints, such as grade 3/4 toxicity, were heavily confounded by pretreatment morbidity, tumour cell type, and disease progression. Response evaluation was confounded by the premature death of patients who were censored, resulting in biased response rates.

The reported absolute absorbed doses in tumorous and nontumorous tissue should be considered with care, since absolute SPECT quantification in the current study was prone to error due to the use of older technology. Although clinical SPECT can be quantitative with errors of less than 10 %, it requires careful set-up and calibration, as well as state-of-the-art SPECT/CT systems and iterative reconstruction software able to accurately model the imaging physics, and to compensate for image-degrading factors (i.e. attenuation, scatter and partial volume) [33].

Work in progress includes optimization of image analysis using SPECT/CT and CT-based attenuation correction (not available at the time), dose-point kernel algorithms, and SPECT-based DVH analysis. Automatic threshold-based segmentation will be refined based on these techniques, to find a balance between the threshold used and the segmented volume. Future studies will focus on defining absorbed dose threshold values for fusion dual-tracer SPECT dosimetry in a prospective controlled setting.

### Conclusion

Fusion TcMAA/TcSC SPECT imaging is a true physiology-based functional imaging tool that reveals dose–response relationships for hepatic RE. Absorbed doses in tumours and in functional liver tissue correlate with response, survival and toxicity in a heterogeneous population. This method may be useful for individualized treatment planning.

## Electronic supplementary material

ESM 1 (DOC 84 kb)

ESM 2 (DOC 51 kb)

ESM 3 (DOC 60 kb)
