# Supplementary material for: Fusion dual-tracer SPECT-based hepatic dosimetry predicts outcome after radioembolization for a wide range of tumour cell types
Source: Eur J Nucl Med Mol Imaging. 2015 Apr 28;42(8):1192–201. doi: 10.1007/s00259-015-3048-z (PMC4480819; doi:10.1007/s00259-015-3048-z)
Supplement: Supplementary file 1 — (DOC 84 kb) [file 259_2015_3048_MOESM1_ESM.doc]

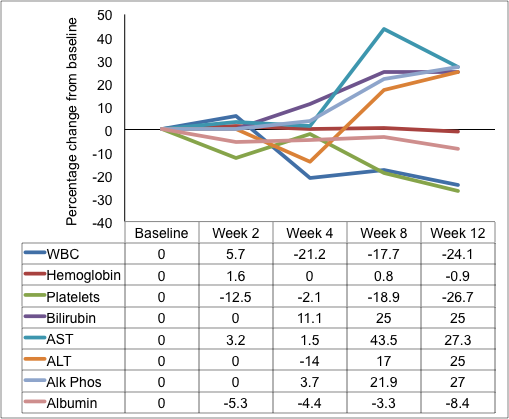


**Supplemental Fig. 1** Median change (%) in laboratory values from baseline, for 111 patients. WBC = white blood cell count, AST = aspartate aminotransferase, ALT = alanine aminotransferase, Alk Phos = alkaline phosphatase.
