# Supplementary material for: Fusion dual-tracer SPECT-based hepatic dosimetry predicts outcome after radioembolization for a wide range of tumour cell types
Source: Eur J Nucl Med Mol Imaging. 2015 Apr 28;42(8):1192–201. doi: 10.1007/s00259-015-3048-z (PMC4480819; doi:10.1007/s00259-015-3048-z)
Supplement: Supplementary file 2 — (DOC 51 kb) [file 259_2015_3048_MOESM2_ESM.doc]

**Supplemental Table 1** Efficacy analysis

| Parameter | Response 3 M | Response 6 M | Survival |
| --- | --- | --- | --- |
| D_T_ (Gy) | **0.026** | 0.069 | **0.004** |
| D_FL-TOT_ (Gy) | 0.106 | 0.177 | 0.413 |
| D_FL-IR_ (Gy) | **0.007** | 0.065 | **0.010** |
| V_T_ (mL) | 0.174 | 0.565 | 0.620 |
| V_FL-IR_ (mL) | 0.143 | 0.065 | 0.060 |
| V_FL-UN_ (mL) | 0.211 | 0.159 | **0.003** |
| V_TOTAL LIVER_ (mL) | 0.327 | 0.121 | 0.732 |
| V_FL-TOT_ (mL) | 0.567 | 0.166 | 0.585 |
| V_FL-IR_ (mL) / V_FL-UN_ (mL) | 0.079 | 0.069 | **0.021** |
| D_T_ (Gy) / D_FL-TOT_ (Gy) | 0.236 | 0.135 | 0.053 |
| D_T_ (Gy) / D_FL-IR_ (Gy) | 0.580 | 0.394 | 0.249 |
| V_T_ (mL) / V_TOTAL LIVER_ (mL) | 0.258 | 0.732 | 0.386 |
| V_FL-UN_ (mL) / V_TOTAL LIVER_ (mL) | **0.042** | 0.055 | **0.003** |
| D_T_ (Gy) = tumour absorbed dose in Gy; D_FL-TOT_ (Gy) = total functional liver absorbed dose in Gy; D_FL-IR_ (Gy) = irradiated functional liver absorbed dose in Gy; V_T_ (mL) = tumour volume in mL; V_FL-IR_ (mL) = irradiated functional liver volume in mL; V_FL-UN_ (mL) = unirradiated functional liver volume in mL; V_TOTAL LIVER_ (mL) = total liver volume in mL; V_FL-TOT_ (mL) = total functional liver volume in mL.  Univariate analysis in 122 Patients. Mann-Whitney non-parametric test for response at 3 and 6 months. Cox regression analysis for survival stratified for primary cell type. P-values are given. Numbers in bold are significant (P-value < 0.05). | | | |
