# Supplementary material for: Fusion dual-tracer SPECT-based hepatic dosimetry predicts outcome after radioembolization for a wide range of tumour cell types
Source: Eur J Nucl Med Mol Imaging. 2015 Apr 28;42(8):1192–201. doi: 10.1007/s00259-015-3048-z (PMC4480819; doi:10.1007/s00259-015-3048-z)
Supplement: Supplementary file 3 — (DOC 60 kb) [file 259_2015_3048_MOESM3_ESM.doc]

**Supplemental Table 2** Toxicity analysis

| Parameter |  | Grade 3 / 4 ^a^ | |  | Grade change ^b^ | REILD ^c^ |
| --- | --- | --- | --- | --- | --- | --- |
| D_T_ (Gy) |  | 0.606 |  | | 0.328 | 0.701 |
| D_FL-TOT_ (Gy) |  | 0.731 |  | | **0.010** | **0.011** |
| D_FL-IR_ (Gy) |  | 0.474 |  | | 0.249 | 0.320 |
| V_T_ (mL) |  | 0.977 |  | | 0.795 | 0.876 |
| V_FL-IR_ (mL) |  | 0.123 |  | | 0.319 | 0.983 |
| V_FL-UN_ (mL) |  | 0.244 |  | | **0.038** | **0.002** |
| V_TOTAL LIVER_ (mL) |  | 0.702 |  | | 0.109 | 0.083 |
| V_FL-TOT_ (mL) |  | 0.688 |  | | 0.094 | **0.047** |
| V _FL-IR_ (mL) / V_FL-UN_ (mL) |  | 0.178 |  | | 0.119 | **0.008** |
| D_T_ (Gy) / D_FL-TOT_ (Gy) |  | 0.362 |  | | 0.305 | 0.174 |
| D_T_ (Gy) / D_FL-IR_(Gy) |  | 0.760 |  | | 0.721 | 0.721 |
| V_T_ (mL) / V_TOTAL LIVER_ (mL) |  | 0.951 |  | | 0.945 | 0.564 |
| V_FL-UN_ (mL) / V_TOTAL LIVER_ (mL) |  | 0.185 |  | | 0.152 | **0.006** |
| D_T_ (Gy) = tumour absorbed dose in Gy; D_FL-TOT_ (Gy) = total functional liver absorbed dose in Gy; D_FL-IR_ (Gy) = irradiated functional liver absorbed dose in Gy; V_T_ (mL) = tumour volume in mL; V_FL-IR_ (mL) = irradiated functional liver volume in mL; V_FL-UN_ (mL) = unirradiated functional liver volume in mL; V_TOTAL LIVER_ (mL) = total liver volume in mL; V_FL-TOT_ (mL) = total functional liver volume in mL.  ^a^ Grade 3 / 4 = absolute grade 3 or 4 toxicity during follow-up.  ^b^ Grade change = maximum change in toxicity grade compared to baseline (range 0-4).  ^c^ REILD = radioembolization-induced liver disease.  Univariate analysis. Kruskal-Wallis 1-way ANOVA non-parametric test for comparison between groups. P-values are given. Numbers in bold are significant (P-value < 0.05). | | | | | | |
